# Severe aortic regurgitation revealing Takayasu arteritis: a case report

**DOI:** 10.1093/ehjcr/ytag569

**Published:** 2026-08-07

**Authors:** Fatima Ezzahra Dommane, Meryem Haboub, Mohamed Khaldi, Abdenasser Drighil

**Affiliations:** Cardiology Department, University Hospital Ibn Rochd, 8, street Lahcen El Arjoun, Casablanca 20100, Morocco; Cardiology Department, University Hospital Ibn Rochd, 8, street Lahcen El Arjoun, Casablanca 20100, Morocco; Cardiology Department, University Hospital Ibn Rochd, 8, street Lahcen El Arjoun, Casablanca 20100, Morocco; Cardiology Department, University Hospital Ibn Rochd, 8, street Lahcen El Arjoun, Casablanca 20100, Morocco

**Keywords:** Takayasu arteritis, Aortic regurgitation, Immunosuppressive therapy, Bentall procedure, Case report

## Abstract

**Background:**

Takayasu arteritis (TA) is a rare chronic autoimmune vasculitis affecting large and medium-sized arteries, mainly in young women, and is more common in Asia. It may cause stenosis, occlusions, aneurysms, aortic dilation, and aortic valve disease. We are reporting a rare case of severe aortic valve insufficiency and aortic dilation in the context of TA.

**Case presentation:**

We report the case of a 39-year-old patient presenting with progressively worsening dyspnoea, associated with fatigue, weight loss, and febrile sensations. Physical examination revealed signs of heart failure (orthopnoea, tachycardia, pulmonary crackles) with a diastolic murmur at the left sternal border and bounding peripheral pulses. The transthoracic echocardiography revealed a severe aortic valve regurgitation with a dilated aortic annulus, slightly thickened cusps, and left coronary cusp prolapse in addition to dilation of the initial aorta confirmed by aortic computed tomography (CT) angiography. Doppler ultrasound of the supra-aortic trunks revealed a regular circumferential hypoechogenic halo-like thickening of the walls of the common carotid arteries and the left subclavian artery suggestive of TA. Positron emission tomography (PET)–CT confirmed active inflammation of the aortic arch. Laboratory tests showed an inflammatory pattern with anaemia, elevated sedimentation rate and C-reactive protein. Immunological workup was negative. Initial management included heart failure management and immunosuppressive therapy with corticosteroids followed by a Bentall procedure. Histopathological examination of the excised aorta confirmed chronic arteritis with aneurysmal changes. The patient demonstrated significant clinical and inflammatory improvement during follow-up.

**Conclusion:**

This case underscores the importance of considering TA in young women with aortic regurgitation and the value of multimodal imaging and multidisciplinary care.

Learning points

- In young women with unexplained severe aortic regurgitation or aortic dilation, Takayasu arteritis must be considered early.
- Multimodal imaging, including PET-CT and vascular Doppler, is key to identifying active inflammation and guiding management.
- Optimal outcomes rely on combined immunosuppressive therapy and timely surgical correction of aortic complication.

## Background

Takayasu arteritis (TA) is a rare granulomatous vasculitis affecting large vessels, particularly the aorta and its branches.^1^

Cardiovascular complications are common and severe. Aortic regurgitation is a major complication, occurring due to aortic root dilation or valve damage.^2^

This case report underscores the importance of early diagnosis and integrated management strategies for TA-related aortic regurgitation (AR) and aortic root dilation.

## Summary figure

**Table:** 

Timeline	Events/Evolution
Hospital admission	39-year-old female for acute decompensated heart failure. Transthoracic echocardiography showed severe AR and dilation of the aorta and left ventricle. Left ventricular ejection fraction of 58% Thoracic CT angiography confirmed dilation of the ascending aorta and the aortic arch According to aetiological investigations and the American College of Rheumatology/European Alliance of Associations for Rheumatology ACR/EULAR 2022 classification, the diagnosis of TA was confirmed Treatment: Heart failure medications, high-dose corticosteroid was initiated to control inflammation, methotrexate was added. Surgery: After 8 months of immunosuppressive therapy and clinical stabilization, the patient underwent a Bentall procedure.
Post operative finding	Follow-up at the post operative period and 1 year later showed that the patient improved clinically with a normal functioning mechanical aortic valve, a non-dilated left ventricle with preserved ejection. Lab tests showed no inflammation.
Take home messages	(i) In young women with unexplained severe AR or aortic dilation, TA must be considered early. (ii) Multimodal imaging, including PET-CT and vascular Doppler, is key to identifying active inflammation and guiding management (iii) Optimal outcomes rely on combined immunosuppressive therapy and timely surgical correction of aortic complications

## Case presentation

We are reporting the case of a 39-year-old female presenting with worsening dyspnoea, fatigue for almost 1 year. She was admitted to the cardiology department for acute decompensated heart failure. On admission, she was apyretic and had a blood pressure of 130/40 mmHg and Corrigan pulse, heart rate of 76 bpm, and oxygen saturation of 96% on room air.

**Figure 1:**
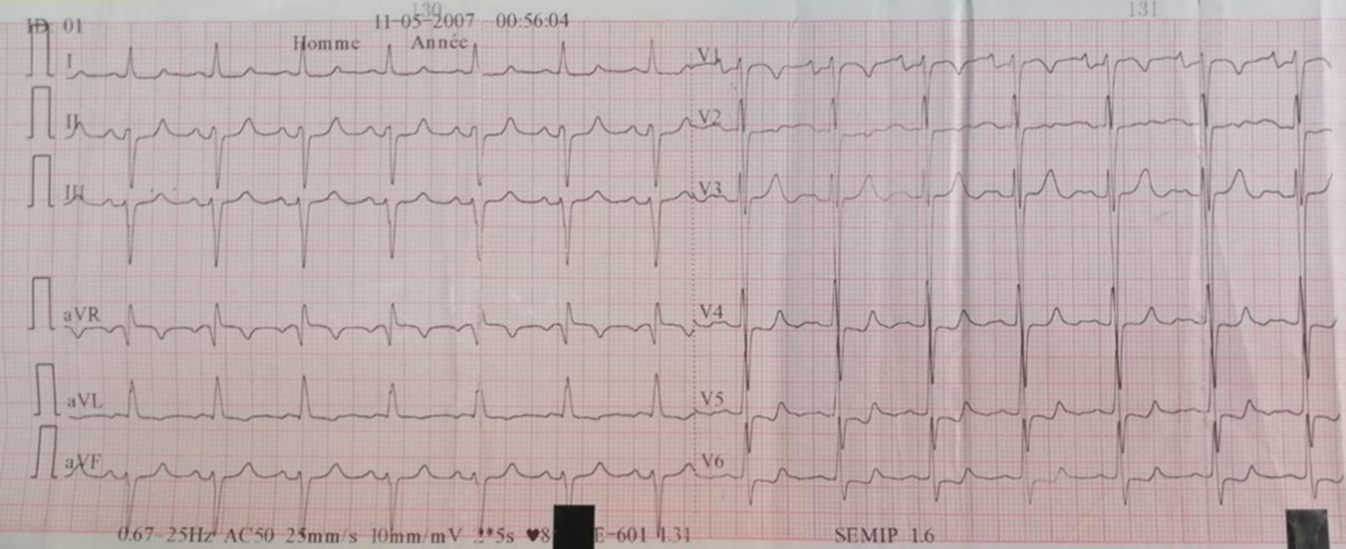
Admission electrocardiogram. Sinus rhythm at 78 beats/min, normal PR interval, normal QRS complexes, with left anterior fascicular block and negative T waves in the lateral leads.

Physical examination found limbs oedema, turgidity of the jugular veins, slight abdominal distension, and decrescendo early-diastolic blowing murmur. The electrocardiogram (*Figure 1*) of our patient showed a regular sinus rhythm at 78 beats/min, with left anterior fascicular block and negative T waves in the lateral leads.

**Figure 2:**
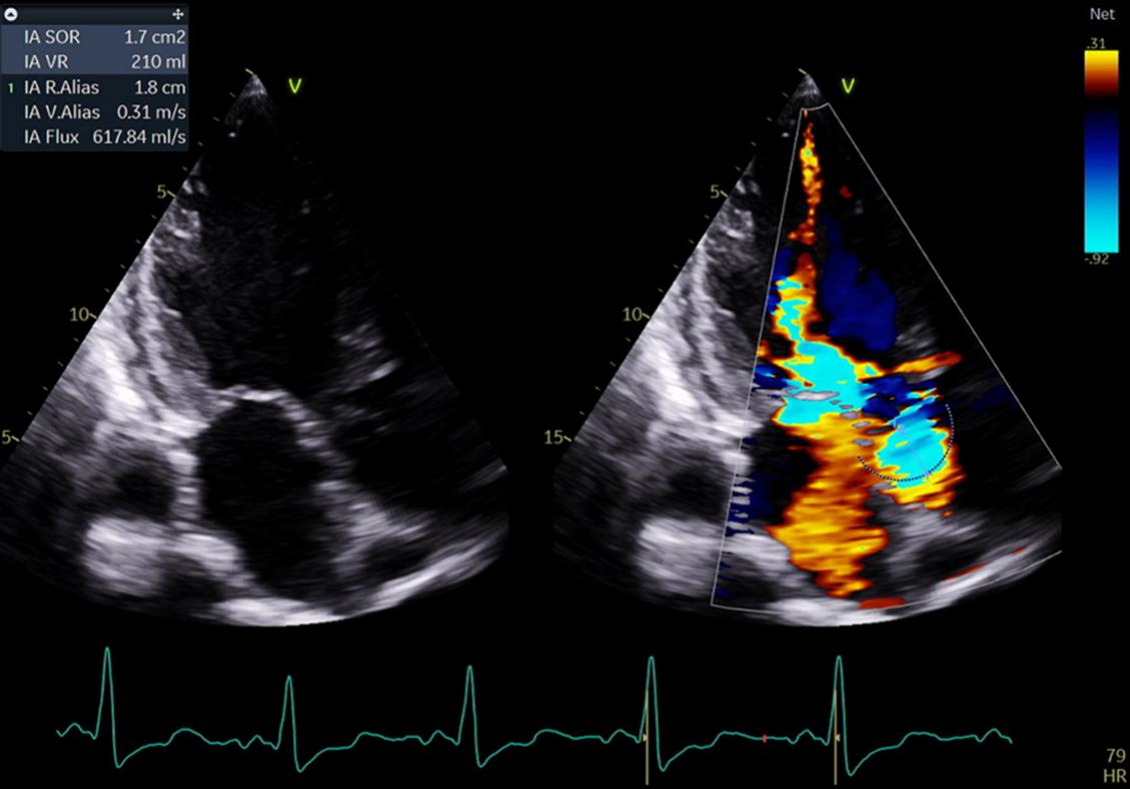
Apical 3-chamber view in proto-diastole. Severe aortic regurgitation due to aortic annulus dilation (aortic annulus = 41 mm) and prolapse of the left coronary cusp. Severity criteria were as follows: proximal isovelocity surface area radius = 18 mm, regurgitant orifice area = 170 mm^2^, regurgitant volume = 210 mL, calculated cardiac output = 11 L/min.

**Figure 3:**
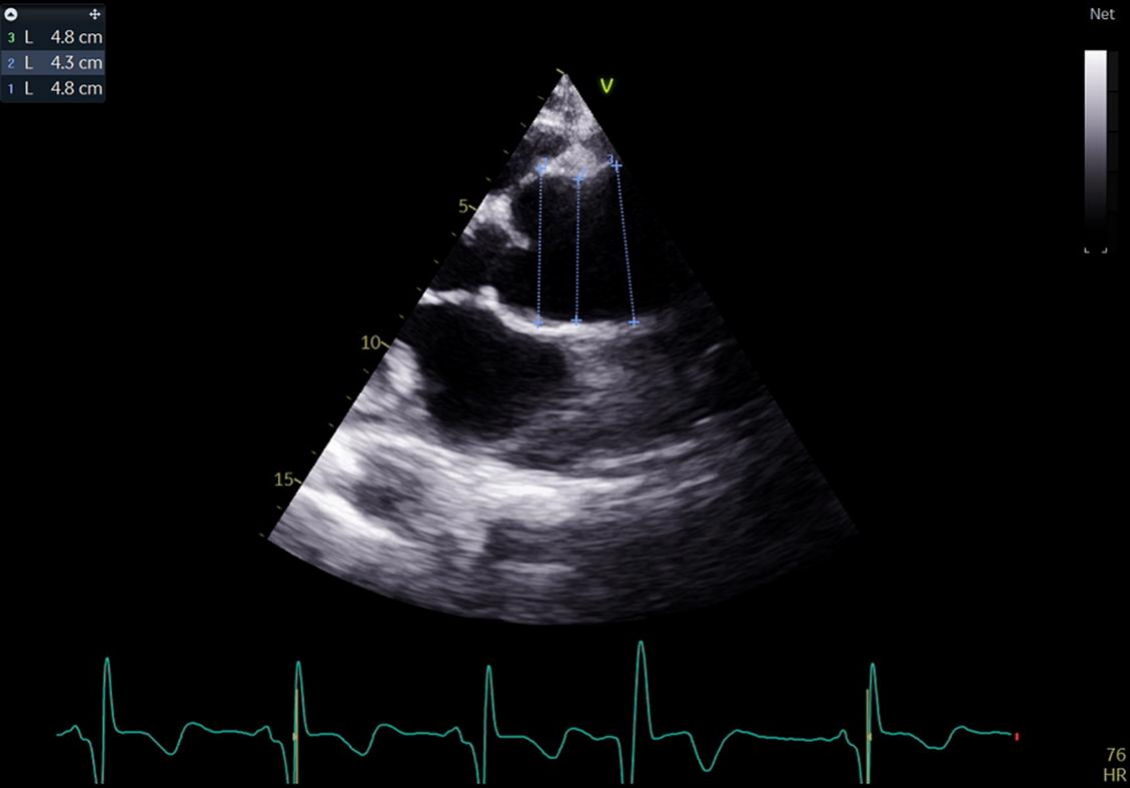
Parasternal long axis view in end-diastole. Dilation of the ascending and horizontal aorta: Valsalva sinuses = 48 mm, tubular junction = 43 mm, ascending aorta = 48 mm.

**Figure 4:**
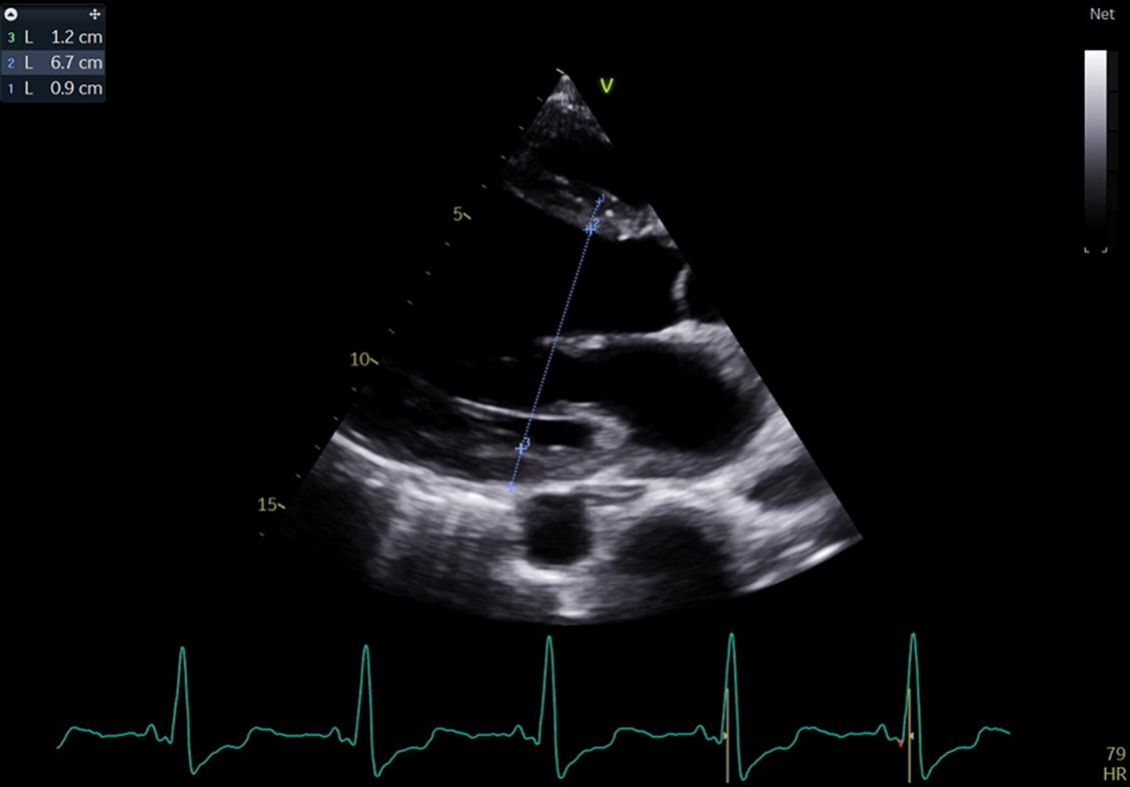
Parasternal long axis view in end-diastole. Dilated left ventricle (left ventricular end-diastolic diameter = 67 mm; 42 mm/m^2^).

**Figure 5:**
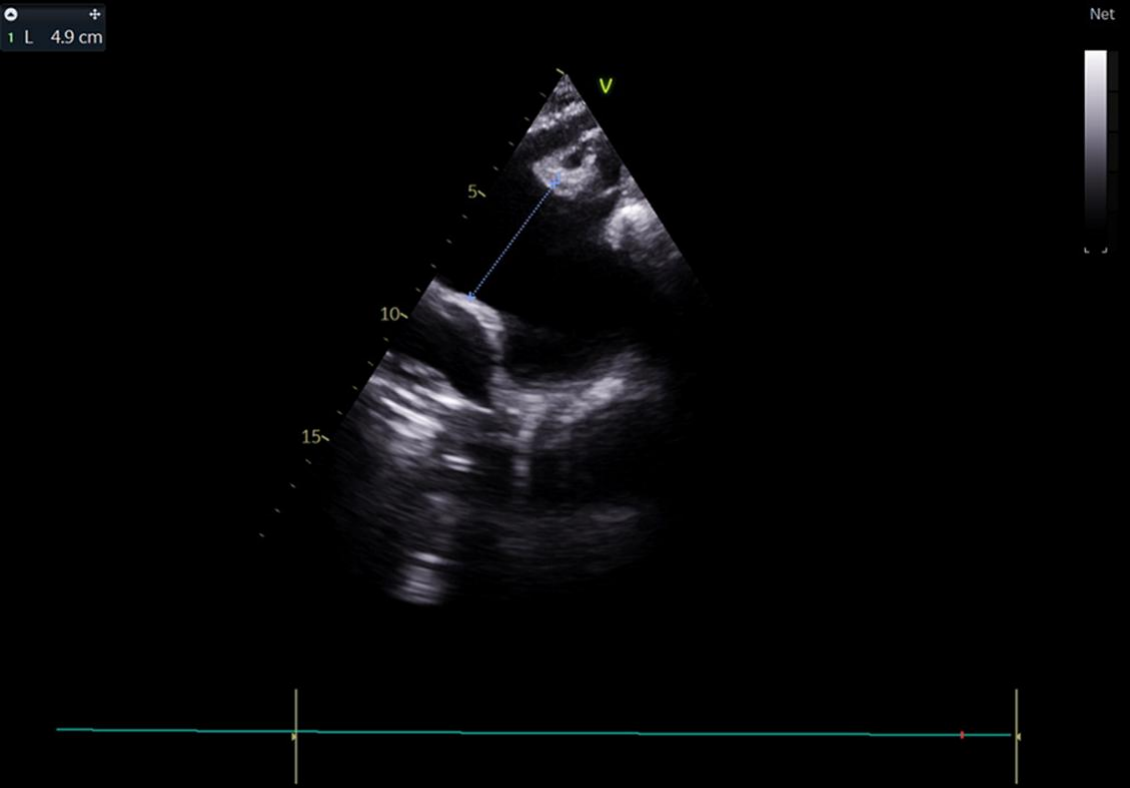
Suprasternal view. Dilation of the aortic arch measuring 49 mm.

The chest X-rays showed cardiomegaly and aortic root dilation. Transthoracic echocardiogram (TTE; *Figures 2–5*) revealed severe AR due to aortic annulus dilation (aortic annulus = 41 mm), prolapse of the left coronary cusp: r proximal isovelocity surface area radius (PISA-r) = 1, 8 cm, regurgitant orifice area = 1, 7 cm^2^, regurgitant volume = 210 mL, cardiac output = 11 L/min, end-diastolic flow velocity in the descending aorta = 28 cm/s, pressure half time 221 ms. Transthoracic echocardiography revealed dilation of the aortic root and thoracic aorta. The aortic annulus measured 41 mm, the sinuses of Valsalva 48 mm, the sinotubular junction 43 mm, and the ascending aorta 48 mm. The aortic arch measured 49 mm, while the descending thoracic aorta measured 29 mm.

Thoracic computed tomography (CT) angiography (see Supplementary file  *S1*) confirmed diffuse dilation of the thoracic aorta with slightly different measurements due to the imaging modality. The aortic annulus measured 40.4 mm, the sinuses of Valsalva 49.7 mm, the sinotubular junction 45.9 mm, the tubular ascending aorta 45.5 mm, and the aortic arch 53.4 mm.

These findings confirmed significant dilation of the aortic root, ascending aorta, and aortic arch.

Duplex ultrasound of the supra-aortic trunks (see Supplementary file  *S2*) revealed a regular circumferential halo-like thickening of the walls of the right and left common carotid arteries and the left subclavian artery, with a maximum thickness of 16, 14, 13 mm, respectively. Biological results showed iron deficiency anaemia (haemoglobin = 9.3 g/dL) ferritin level of 11 ng/mL, erythrocyte sedimentation rate (ESR) = 119 mm/hC-reactive protein = 82 mg/L. Syphilis serology, antinuclear antibodies, Coombs test, and rheumatoid factor were negative. F-fluorodeoxyglucose positron emission tomography (FDG-PET) scan confirmed active inflammation of the aortic arch (see Supplementary file  *S3*). The findings were consistent with TA and fulfilled the American College of Rheumathology/European Alliance of Associations for Rheumatology (ACR/EULAR) 2022 classification criteria^3^: The two absolute criteria were present (age <60 years, evidence of vasculitis on imaging) with 5 points on additional criteria (female sex: +1 point; involvement of four vascular territories: +3 points; symmetric involvement of paired arteries: +1 point; see Supplementary file  *S4*). In our patient, several aetiologies were excluded, including infectious causes such as syphilitic aortitis. Autoimmune conditions were also considered, and the immunological workup including antinuclear antibodies and rheumatoid factor was negative. The young age of the patient, the pattern of vascular involvement affecting multiple large vessels, and the imaging findings of circumferential arterial wall thickening and PET–CT evidence of vascular inflammation strongly supported the diagnosis of TA.

We conducted a medical therapy based on oral furosemide 40 mg twice daily, in addition to potassium supplementation, ramipril 2.5 mg once daily, spironolactone 50 mg daily, and iron supplementation. Prednisone 1 mg/kg/day was initiated to control inflammation. Aspirin 100 mg and atorvastatin 10 mg daily were added to prevent accelerated atherosclerosis. Methotrexate was added.

Following initiation of immunosuppressive therapy with high-dose corticosteroids and methotrexate, the patient demonstrated significant clinical improvement with regression of heart failure symptoms. Inflammatory markers also improved significantly, with C-reactive protein (CRP) decreasing from 82 mg/L at admission to 4.7 mg/L during follow-up.

After 8 months of immunosuppressive therapy and clinical stabilization, the patient underwent a Bentall procedure. The pathological examination of the surgical specimen confirmed the presence of an aneurysmal lesion in a pre-dissection state. Histological analysis demonstrated chronic inflammatory arteritis with fibrous thickening of the arterial wall and structural remodelling of the vascular layers, compatible with large-vessel granulomatous vasculitis consistent with TA. On post-operative follow-up, she improved clinically, with a normal functioning mechanical aortic valve and a non-dilated left ventricle with preserved ejection fraction (see Supplementary file  *S5*). The CRP was less than 6 mg/L.

## Discussion

Takayasu arteritis, also known as the ‘pulseless disease’^4^ is a rare granulomatous panarteritis affecting large vessels, particularly the aorta and its main branches.^5^ It affects young women, especially of Asian descent.^4^ The primary pathological feature is the extensive destruction of medial elastic fibres, which are essential for maintaining the aortic wall’s strength. Early TA shows granulomatous inflammation with cellular infiltration; later scarring causes fibrotic intimal thickening, leading to aortic and coronary stenosis with possible residual inflammation.^5^ Aortic regurgitation is a severe complication of TA. It has been reported as a complication of up to 25% of TA cases.^6^ It represents unique diagnostic and therapeutic challenges and mortality.^7,8^ It arises from specific vascular changes including^2^ aortic root dilation leading to valve leaflet malcoaptation with cusp thickening, retraction, or perforation.^9^ Severe AR causes left ventricular volume overload, progressive dilation, and heart failure. Multimodality imaging (CT, magnetic resonance imaging [MRI], 18-FDG-PET–CT) is essential to assess AR and vascular complications.^10^ Transthoracic echocardiography and transoesophageal echocardiography are first-line investigations for assessing the aetiology, mechanism, severity, and cardiac impact of the AR and identifying associated aortic root dilation. In this case, TTE identified a severe AR due to aortic annulus dilation and a prolapse of the left coronary cusp associated with a dilation of the ascending aorta and the aortic arch. Computed tomography or MRI angiography is essential to confirm aortic wall thickening, stenosis, or aneurysmal changes. In addition, MRI allows free choice of imaging planes as prescribed by the scan operator; in addition to assessing the severity of the AR, it is able to quantify cardiac remodelling and provide insight into the mechanism of regurgitation.^10^ 18-FDG-PET-CT detects active inflammation in the arterial walls not only in patients with active TA before treatment but also in relapsing patients receiving immunosuppressive agents.^11^ A Japanese study showed the role of PET-CT that reveals the disease activity directly; in fact, the general inflammatory markers did not necessarily coincide with disease activity shown by 18-FDG-PET-CT.^12^ In our case, PET-CT demonstrated active arterial wall inflammation in the aortic arch and so established the diagnosis of aortitis. The findings were consistent with TA and fulfilled the 2022 ACR/EULAR classification criteria.^3^ Laboratory tests showed elevated ESR and CRP, negative autoantibodies (e.g. antinuclear antibodies [ANA], rheumatoid factor [RF]), distinguishing TA from other autoimmune diseases.^13^ The treatment of TA-related AR requires a multidisciplinary approach involving cardiologists, internists, and surgeons for optimal outcomes. Persistent active inflammation leads to cardiovascular complications. Optimal treatment could prevent these complications. Medical management requires corticosteroids as first-line therapy (e.g. prednisone 1 mg/kg/day) to suppress active inflammation, immunosuppressants such us methotrexate or azathioprine as steroid-sparing agents, and biotherapy (e.g. tocilizumab) for refractory cases. Prednisolone was prescribed for 4 to 6 weeks after control of inflammation, followed by tapering. Methotrexate, azathioprine, mycophenolate mofetil, Anti-TNFα or anti-IL-6 therapies, such as tocilizumab, may be required in persistent inflammation.^14^ The regular monitoring of inflammatory markers is necessary to guide therapy.^15^ In severe AR with symptoms or left ventricular dysfunction, the surgical intervention can be aortic valve replacement (AVR) or composite graft replacement (CGR) where the aortic valve is replaced along with the aortic root. The timing of surgery is critical, as active inflammation increases perioperative risks. Immunosuppression is typically optimized before surgery. The aortic root surgery may be required for patients with significant dilation or aneurysmal changes.^16^ A study determined the late outcome of surgical treatment for AR due to TA and correlated it with evidence of inflammation on pathological examination. Late outcomes are favourable, but active inflammation increases aneurysm risk. Both AVR and CGR show outcomes;^6^ valve-sparing repair is discouraged due to multimodality recurrent regurgitation. A CGR may provide more durable results than AVR. In Osamu *et al*.’s series, four underwent AVR and the others received aortic root replacement. Two AVR patients later required a CGR due to aortic root aneurysm and prosthetic valve detachment, 12 and 7 years postoperatively.^17^ A CGR is preferred over AVR when the aortic root diameter exceeds 45 mm or if it is considered fragile during the perioperative period.^16^ After surgery, continued immunosuppression is necessary to prevent disease relapse and protect surgical outcomes. Patients with TA require lifelong follow-up to monitor for relapse, progression of vascular disease, or prosthetic valve dysfunction. The combination of medical and surgical treatment has improved the survival and quality of life in these patients.^6^ The prognosis of TA-related AR depends on early diagnosis and effective disease control. Thus, the most frequent operation performed now on patients with TA is AVR.^13^

## Conclusion

Takayasu arteritis, even when rare, demonstrates through AR how autoimmune vasculitis can cause structural cardiac damage. Our case underscores that early recognition, tailored immunosuppressive and surgical therapy, and vigilant monitoring are essential. Personalized treatment and coordinated multidisciplinary care remain key to improving long-term outcomes and ensuring optimal patient management.

## Supplementary Material

ytag569_Supplementary_Data

## Data Availability

No new data were generated or analysed in support of this article.

## Lead author biography

Doctor Fatima Ezzahra Dommane, Medical Doctor of Cardiology in the Faculty of Medicine, Hassan II University Casablanca, Morocco.

## Notes

**Consent:** The authors confirm that written consent for submission and publication of this case report including images and associated text has been obtained from the patient in line with COPE guidance.
