# Supplementary material for: Severe aortic regurgitation revealing Takayasu arteritis: a case report
Source: Eur Heart J Case Rep. 2026 Aug 7;10(8):ytag569. doi: 10.1093/ehjcr/ytag569 (PMC13472166; doi:10.1093/ehjcr/ytag569)
Supplement: Supplementary file 1 [file ytag569_supplementary_data.zip › Supplementary figures.docx]

**Fig S1:** Thoracic CT angiography Transverse View

Dilation of Aortic arch at 53,4 mm and Valsalva sinuses at 49,7 mm

Thoracic CT angiography [Sagittal View](https://www.google.com/search?q=sagittal+view&client=opera&sca_esv=90ae87e741ae3c67&sxsrf=AE3TifMroLO-xuGuSRyxK1C7Oq3HZ2vLsw%3A1764424750894&ei=LvwqaeCXNoio9u8PsIKN0Ao&ved=2ahUKEwjjhK-3wpeRAxV8gf0HHYc5MHAQgK4QegQIAhAB&uact=5&oq=coupe+sagittale+scanner+thoracique+anglais&gs_lp=Egxnd3Mtd2l6LXNlcnAiKmNvdXBlIHNhZ2l0dGFsZSBzY2FubmVyIHRob3JhY2lxdWUgYW5nbGFpczIFECEYoAEyBRAhGKABSOgTUMsDWK0ScAF4AZABAJgBxwGgAYYLqgEDMC44uAEDyAEA-AEBmAIJoALpC8ICChAAGLADGNYEGEfCAgYQABgWGB7CAggQABiABBiiBMICBBAhGBXCAgUQIRifBcICBxAhGKABGAqYAwCIBgGQBgiSBwUxLjYuMqAHviKyBwUwLjYuMrgH4gvCBwcwLjEuNC40yAc7&sclient=gws-wiz-serp&mstk=AUtExfDsl0hwxRPp8k_qBijhuNpZZ8WP09q6a93NQIhszixYi5QfFHJbfBOE9cq5KaWxoRnUIk_csMFxAiby_aw37br4H9k7kb4SAKhUlwy5rszgo3RRfoVOqrCQseIXbUkznqTGrK8CxKCyM5xyUW3My7BRGZv5hBkixkABC6rAfyP-_C_KuV7BHkJJwbCMrrHHUhdXuLvBcNa2Ga2FA28v2I3ppa9d5VTGtyD9l5EY3nOsf6Z7kWkTIDJTG5HEeb5ytMJBVo3p27ptcHiJ_nVCT2jh&csui=3)

Dilation of the Ascending aorta

**Fig S2:** Doppler ultrasound of the supra-aortic trunks

Regular circumferential hypoechogenic halo-like thickening of the walls of the common carotid arteries and the left subclavian artery suggestive of Takayasu arteritis

**Fig S3:** PET-CT

Active inflammation of the aortic arch

**Fig S4:** ACR/EULAR 2022 criteria

Confirmed Takayasu Arteritis Diagnosis

**Fig S5: Post operative TTE**

Normal functioning mechanical aortic valve and non-dilated left ventricle with preserved ejection fraction (LVEF = 55%)
