# Supplementary material for: Severe aortic regurgitation revealing Takayasu arteritis: a case report
Source: Eur Heart J Case Rep. 2026 Aug 7;10(8):ytag569. doi: 10.1093/ehjcr/ytag569 (PMC13472166; doi:10.1093/ehjcr/ytag569)
Supplement: Supplementary file 1 [file ytag569_supplementary_data.zip › Supplementary video legends.docx]

**Supplementary Video Legends:**

**Figure S6 Video 1**

Parasternal long axis view: Severe aortic regurgitation

**Figure S7 Video 2**

Apical five chambers: Severe AR

**Figure S8 Video 3**

Short parasternal axis: Severe AR

**Figure S9, S10, S11 Video 4,5,6**

Post operative TTE: Normal functioning mechanical aortic valve and non-dilated left ventricle with preserved ejection fraction (LVEF = 55%)
